# The role of blogs and news sites in science communication during the COVID-19 pandemic

**DOI:** 10.3389/frma.2022.824538

**Published:** 2022-09-23

**Authors:** Grischa Fraumann, Giovanni Colavizza

**Affiliations:** ^1^R&D Department, TIB – Leibniz Information Centre for Science and Technology, Hannover, Germany; ^2^Centre for Science and Technology Studies (CWTS), Leiden University, Leiden, Netherlands; ^3^Institute for Logic, Language and Computation (ILLC), University of Amsterdam, Amsterdam, Netherlands

**Keywords:** altmetrics, knowledge transfer, science communication, science and society, blogs, news sites, COVID-19, scholarly information

## Abstract

We present a brief review of literature related to blogs and news sites; our focus is on publications related to COVID-19. We primarily focus on the role of blogs and news sites in disseminating research on COVID-19 to the wider public, that is knowledge transfer channels. The review is for researchers and practitioners in scholarly communication and social media studies of science who would like to find out more about the role of blogs and news sites during the COVID-19 pandemic. From our review, we see that blogs and news sites are widely used as scholarly communication channels and are closely related to each other. That is, the same research might be reported in blogs and news sites at the same time. They both play a particular role in higher education and research systems, due to the increasing blogging and science communication activity of researchers and higher education institutions (HEIs). We conclude that these two media types have been playing an important role for a long time in disseminating research, which even increased during the COVID-19 pandemic. This can be verified, for example, through knowledge graphs on COVID-19 publications that contain a significant amount of scientific publications mentioned in blogs and news sites.

## Introduction

Blogs are important channels for dissemination of research (Woolston and Brown, 2018; Ross-Hellauer et al., 2020) and are increasingly created by various organizations, such as learned societies, universities, departments, and individual researchers. Blogging about research is considered as part of outreach activities by researchers, universities, and research-performing organizations (Cameron et al., 2016; Alperin et al., 2019). This could be influenced by the fact that higher education institutions are usually evaluated to a certain extent based on their online visibility (Aguillo et al., 2008; Belli and Gonzalo-Penela, 2020). Furthermore, media mentions of scholars are considered as an additional indicator for some rankings in higher education, particularly in specific academic disciplines, such as economics and business studies (BMBF, 2018). Discussions on the values and limits of these rankings are outside the scope of this review.

However, a vast amount of content is generated in blogs. As of January 2022, WordPress users generated 70 million new blog posts and 77 million new comments each month and “over 409 million people view more than 20 billion (WordPress) pages each month” (WordPress, 2022). WordPress is only one possible way to publish blogs among many other blog publishing tools, and it is an illustration of the wide use of blogs. In addition to blogs, news sites play an important role in science communication particularly by the typical mentioning of research outputs by journalists (NAS, 2017).

In this mini review, we frame blogs and news sites as scholarly knowledge transfer channels for the general public. This topic gains importance due to the ongoing COVID-19 pandemic, as of January 2022 (WHO, 2022). Due to the far-reaching impact of research on the pandemic and vice-versa, it is, therefore, crucial to provide an example of how a fraction of knowledge on this essential topic is transferred online to the wider public. Such an analysis can be of relevance for several stakeholders, for example, researchers from various academic disciplines, science communicators, policymakers, and the wider public. Thus, the purpose of this review is to provide an overview on COVID-19 research and two altmetric sources as science communication outlets, namely blogs and news sites. To put blogs and news sites as altmetric sources into context, we also reviewed literature that had been published before the COVID-19 pandemic. We selected literature from relevant journals, conferences, and workshops in scientometrics, and enriched this review with further reports and websites on the topic.

## An overview on blogs and news sites as altmetric sources

Altmetric aggregators track the interaction of diverse audiences with research outputs across various data sources, such as online social networks (OSNs), news sites, and policy documents. Initially proposed in the so-called Altmetrics Manifesto in 2010 by Priem et al. (2010), these online events are nowadays captured and collected by altmetric data aggregators. *Altmetric.com* is one of the largest altmetric data aggregators in terms of its use in scientific publications and studies. Other aggregators include *Crossref Event Data (CED), PlumX*, and the *US Public Library of Science's (PLOS) article-level metrics (ALM)*, while other open-source projects developed visualizations of altmetrics (Hauschke et al., 2018).

Curated lists of blogs are tracked by several altmetric data aggregators, for example, Altmetric.com and PlumX. There is a distinction between blogs and microblogs, which refers to online social networks such as Twitter (Bornmann and Haunschild, 2018). Surprisingly, due to the high amount of news that is shared on Twitter, the online social network was even considered as news media itself (Kwak et al., 2010). Scholars have also investigated how news coverage of research outputs is related to citation rates, for example, in specific academic disciplines, such as biomedicine (Dumas-Mallet et al., 2020).

## Altmetrics as knowledge transfer channels

In general, medicine belongs to one of the main subject categories in Altmetric.com, CED, and PlumX with regards to news and blog sources (Ortega, 2020). As this subject also includes research on COVID-19, it might be promising to study blogs and news sites as knowledge transfer channels. Altmetrics as knowledge transfer channels were discussed at an international conference in 2019, and we adopted this term for this review (Ortega and Esquinas, 2019).

The similarity of topics from scientific publications (e.g. in the research area of Big Data) that are mentioned across various Altmetric.com sources, such as in blogs and news sites, is another area of interest for scholars (Lyu and Costas, 2021). Additionally, a study has been carried out to calculate the time that it takes until a registered Digital Object Identifier (DOI) from the bibliographic database *Crossref* is reported for the first time in an Altmetric.com source. Blogs and news sites seem to be among the altmetric sources, where such a report happens relatively fast compared to other sources, that is “within the first few days after publication” (Fang and Costas, 2020).

There might be multiple reasons for mentioning a research output in a blog post and a news article, and these reasons can even differ within the same blog or news site. For example, the research outputs mentioned in the news media based on their value is a research topic in journalism studies (Badenschier and Wormer, 2012; Tunger et al., 2018; Prados-Bo and Casino, 2021). Another topic of interest is how users select news and how this selection can be influenced (van der Sluis et al., 2012). The way in which the media reports about COVID-19 is a topic of interest in several studies, for example, to find out how preprint research is described by news outlets (Fleerackers et al., 2021).

## The relation of blogs and news sites as altmetric sources

Furthermore, the coverage of different altmetric data aggregators varies, and they track different lists of blogs and news sites. Due to such differences among altmetric data aggregators, their coverage has been compared in several research projects (Zahedi and Costas, 2018). Generally speaking, blogs and news sites are closely related, for example, the content that is used in blogs often stems from news sites (Nuernbergk, 2014). There are also several examples, where a certain topic was first discussed on a blog, and later picked up by news sites, or where bloggers fact checked the content of news sites (Scott, 2008), an evaluation that is also called gatewatching (Bruns, 2016). While this led to a transformation of how news are reported, some hurdles still exist (Bruns, 2016). For example, bloggers need to set up their own blog or join an existing blog as an author (Bruns, 2016). Another requirement is to constantly provide new blog posts for a longer period (Bruns, 2016). What is more, the distinction between blogs and news sites is not always crystal clear, as blogs that report news are also sometimes called news blogs (Bruns, 2016). Furthermore, blogs are not a genre as such, but rather consist of several sub-genres (Lomborg, 2009). Another type of blog only disseminates articles of interest from other sources (Bruns, 2016), and such types of blogs are typically also included as altmetric sources. Concerning blogs, evaluation of other sources and dissemination of information seems to be more common practices than reporting (Bruns, 2016). News sites also exchange articles with each other. Going back in time, this exchange started as early as in the seventeenth century, when the first printed gazettes in Europe developed a way of exchanging information (Colavizza et al., 2015).

Considering the relevance of blogs, a content analysis of these altmetric sources has been suggested by scholars (Shema et al., 2015; Barata, 2018; Fraumann, 2020), an approach that is also used to study other altmetric sources, such as Twitter (Araújo, 2020). This approach entails the identification of communities of attention (Haustein et al., 2015), that is, a network of users that engage with a research output online, for example, by retweeting it. Several studies have been carried out to unveil the underlying data on blogs that also contribute to altmetric counts (Shema et al., 2014, 2015; Fraumann et al., 2015; Barata, 2018).

Additionally, another study compared the mentions of publications on blogs and news sites among three altmetric data aggregators, namely PlumX, Altmetric.com, and Crossref Event Data (CED) (Ortega, 2020). CED also provides an Application Programming Interface (API) that may be used by others to build their own scientometric data services (Hauschke et al., 2018). Publications on blogs and news sites seem to be mentioned less frequently compared to other altmetric sources, and Altmetric.com has the highest coverage compared to the other data aggregators (Ortega, 2020). The variance between altmetric data aggregators in altmetric sources, such as blogs and news sites, fuels an ongoing debate in scientometrics (Bar-Ilan et al., 2019). Unlike PlumX and CED, Altmetric.com seems to be the only one that captures older events, that is, links to blogs as early as 2005 (Ortega, 2019). Data aggregators, such as Altmetric.com, decide on the sources to be included in their coverage (Meschede and Siebenlist, 2018), and thus, control the information flow as gatekeepers, which can also be observed in other networks (Belardinelli, 2019). Nevertheless, Altmetric.com also provides the possibility for users to suggest news sites, blogs, public Facebook pages, and YouTube channels as additional data sources via an online form^1^, as of 17 January 2022. According to Tunger et al. (2018), there is a need to include more altmetric sources that relate to economy or policy areas.

## Availability of data sources during the COVID-19 pandemic

Our literature review focused on studies that discuss blogs and news sites as altmetric sources. The global health crisis led to new research initiatives from several disciplines. For example, public datasets are being studied, such as publications on COVID-19 and the previous coronavirus publications from a scientometric perspective (Porter et al., 2020; Colavizza et al., 2021); existing knowledge graphs are being extended (Turki et al., 2021); or sentiment analysis on science news are being conducted (Ferreira et al., 2020). Meta-research on COVID-19 is considered as an emerging research field, given its impact on research and researchers around the world (Colavizza, 2021). In general, the response of academia to public health crises is a research topic in scientometrics (Zhang et al., 2020), while the role of open access and international scientific collaboration during such emergencies has been discussed in several studies (Belli et al., 2020; Homolak et al., 2020). Strikingly, 210,183 publications on COVID-19 have been reported in the bibliographic database *Scopus*, as of 1 August 2021 (Ioannidis et al., 2021).

On top of that, the mentions of research publications in news sites and blogs, among other altmetric sources, have been extensively analyzed during the COVID-19 pandemic, for example, to make use of the smaller time window compared to citation counts (Boetto et al., 2021). Boetto et al. (2021) conclude based on PlumX data that blogs and news sites are two of the altmetric sources that can be used to detect early scholarly communication on COVID-19, in addition to Twitter and Facebook. Furthermore, Colavizza et al. (2021) analyzed the COVID-19 Open Research Dataset (CORD-19) from a scientometric perspective on 1 July 2020, a dataset of 169,821 COVID-19 and coronavirus publications. The authors identified news (222,996 mentions) and blogs (29,119 mentions) as the sources that directly come after Twitter (5,868,992 mentions), when COVID-19 publications are mentioned on the Internet. The higher mentions of publications in news compared to blogs might also signal the importance of the topic for the wider public.

Compared to the other above-mentioned altmetric data aggregators, Altmetric.com seems to have the highest coverage in blogs and news sites, among others (Ortega, 2018). Given the limited space of this review, we provide only an overview of this dataset. Still, we would like to stress that a comparison of various data aggregators is instructive, such as conducted, for example, by Ortega (2018, 2019, 2020). Altmetric.com has included blogs as a category since October 2011 (Altmetric.com, 2020a), and it tracks over 15,000 academic and non-academic blogs via Rich Summary Site (RSS) feeds (Altmetric.com, 2020a,c). This number also changes depending on how many blogs are curated by Altmetric.com. The blogs are updated daily, and if a link to a ‘scholarly output' appears in a blog, the blog is harvested (Altmetric.com, 2020c).

News sites have been harvested by Altmetric.com since October 2011, and a new retrieval process has been in place since December 2015 (Altmetric.com, 2021a). News sites are also called mainstream media outlets by Altmetric.com (Altmetric.com, 2020b). Altmetric.com regularly harvests over 5,000 English and non-English news outlets (Altmetric.com, 2020a,b). Similar to the list of curated blogs, this number also changes regularly based on the curation efforts by Altmetric.com, for example, if users suggest new sources. This curated list is harvested in real-time by an unknown third-party provider directly via APIs or RSS feeds (Altmetric.com, 2020c, 2021b), although the full list cannot be downloaded directly from the public website. Hence, it is suggested to use the API to retrieve this list. The third-party data provider searches for direct hyperlinks to scholarly outputs in news articles. Furthermore, Altmetric.com uses text mining to search the news articles for mentions of scholarly outputs. To be tracked, the news article “must include at least the name of an author, the title of a journal, and a publication date” which is then matched with metadata in the Crossref API (Altmetric.com, 2020d). There is a difference between the harvesting frequency of these sources. News sites are harvested in real-time, while blogs are only harvested daily (Altmetric.com, 2021b).

In addition to the reviewed literature on blogs and news sites above, we conducted a query in the COVID-19 Altmetric.com in-house database from the Center for Science and Technology Studies (CWTS). The data from Altmetric.com used in this query is updated up to 21 January 2021. The database was created at the beginning of the COVID-19 pandemic in 2020. The related Altmetric.com in-house database is described in more detail in previous studies, for example, by Lyu and Costas (2021). We counted the overall amount of blogs and news sites in the database. The query resulted in 541,649 news and 63,288 blog mentions, totalling 604,937 mentions in both altmetric sources. This leads us to conclude that there is a high amount of mentions of COVID-19 related publications in blogs and news sites.

Apart from the specific COVID-19 CWTS Altmetric database, we also queried the total number of publications that were tracked on blogs and news sites in the overall CWTS Altmetric database. The World Health Organization (WHO) characterized COVID-19 as a pandemic in a media briefing on 11 March 2020 (WHO, 2020). We consider this media release as an event that might have increased science communication on several online sources, including on blogs and news sites. From 10 March 2019 until 10 March 2020, there were 104,547 unique publications tracked on blogs and 177,697 unique publications on news. From 11 March 2020 until 11 March 2021, these amounts increased by around 12% to 116,650 unique publications on blogs, and by around 19% to 211,828 unique publications on news sites, respectively. As such, we conclude that the number of publications that were tracked by Altmetric.com on blogs and news sites increased slightly during this period. However, it is impossible to test if this increase would have also happened within the same year without a pandemic.

## Further available data sources

Obviously, in addition to Altmetric.com, there are several other news tracking services, although Altmetric.com provides some different features, such as linking to the research outputs themselves (Altmetric.com, 2020e). For example, apart from the well-known *Google News*, there is also the *European Media Monitor*, which tracks “thousands of news sources in over 70 languages”, is updated every 10 minutes, and can be followed via RSS feeds. The service was developed by the European Commission's Joint Research Center (EC, 2022). Further studies retrieve news coverage of publications from the *Factavia* database that provides access to global news, among others (Prados-Bo and Casino, 2021). Furthermore, how news are presented in *Google Search* or other search engines is a research field in itself (Ørmen, 2016). Another source of interest in the altmetrics sphere with regards to news is *EurekAlert!*, a press release aggregator by the American Association for the Advancement of Science (AAAS). This particular news site is also studied as part of the Altmetric.com portfolio (Bowman et al., 2019). The advantage of the Altmetric.com dataset is that it includes blogs and news sites in one file, which can be imported to and analyzed in relational databases, such as Structured Query Language (SQL). Still, we suggest analyzing various data providers to get a broader picture of blogs and news mentions.

## Discussion

Taking into account previous studies, we argue that blogs and news sites have always been playing an important role in disseminating research, and this role became more prominent during the COVID-19 pandemic. We consider a direct comparison between publications before the COVID-19 pandemic and during this global health crisis as infeasible since this event is a disruptive phenomenon (Fassin, 2021). While it will only be possible to understand the whole picture of the role of blogs and news sites in disseminating research on COVID-19 after the end of the pandemic, the sheer amount of reported publications on COVID-19 calls for a brief overview of this topic which we provided here. We expect that researchers and practitioners from various disciplines as well as science communicators, policymakers, and the wider public can benefit from such an overview. While we mainly focused on blogs and news sites as altmetric sources due to the limited space of this mini review, several other sources can be considered for further reviews on the role of knowledge transfer channels during the COVID-19 pandemic. We would also suggest to study the amount of altmetrics sources that have been added during the pandemic by altmetric data aggregators.

What is more, how public health topics are captured by blogs and news media needs to be evaluated on an ongoing basis. In particular, when new global crises emerge, this topic is essential to study science communication from a different angle. How will the role of certain blogs and news sites change? What role will these two altmetric sources have in the future? We only covered a minor aspect of this research area and encourage other researchers to continue on this path.

## Author contributions

All authors listed have made a substantial, direct, and intellectual contribution to the work and approved it for publication.

## Funding

This research was funded by the German Federal Ministry of Education and Research (BMBF) under grant number 01PU17019.

## Conflict of interest

The authors declare that the research was conducted in the absence of any commercial or financial relationships that could be construed as a potential conflict of interest.

## Publisher's note

All claims expressed in this article are solely those of the authors and do not necessarily represent those of their affiliated organizations, or those of the publisher, the editors and the reviewers. Any product that may be evaluated in this article, or claim that may be made by its manufacturer, is not guaranteed or endorsed by the publisher.

## Footnotes

^1^ https://altmetric.typeform.com/to/CBfb1D?typeform-source

## References

[B1] Aguillo I. F. Ortega J. L. Fernández M. (2008). Webometric ranking of world universities: introduction, methodology, and future developments. Higher Educ. Eur. 33, 233–244. 10.1080/03797720802254031

[B2] Alperin J. P. Muñoz Nieves C. Schimanski L. Fischman G. E. Niles M. T. McKiernan E. C. (2019). Meta-research: How significant are the public dimensions of faculty work in review, promotion and tenure documents? eLife 2019 8, e42254. 10.7554/eLife.42254 30747708 PMC6391063

[B3] Altmetric.com (2020a). Attention Sources Tracked by Altmetric. Available online at: https://help.altmetric.com/support/solutions/articles/6000235983-attention-sources-tracked-by-altmetric (accessed January 17, 2022).

[B4] Altmetric.com (2020b). News and Mainstream Media. Available online at: https://help.altmetric.com/support/solutions/articles/6000235999-news-and-mainstream-media (accessed January 17, 2022).

[B5] Altmetric.com (2020c). Blogs. Available online at: https://help.altmetric.com/support/solutions/articles/6000235927-blogs (accessed January 17, 2022).

[B6] Altmetric.com (2020d). Text Mining. Available online at: https://help.altmetric.com/support/solutions/articles/6000240263-text-mining (accessed January 17, 2022).

[B7] Altmetric.com (2020e). Altmetric and Media Monitoring. Available online at: https://help.altmetric.com/support/solutions/articles/6000234231-altmetric-and-media-monitoring (accessed January 17, 2022).

[B8] Altmetric.com (2021a). Attention Sources Coverage Dates. Available online at: https://help.altmetric.com/support/solutions/articles/6000240455-attention-sources-coverage-dates (accessed January 17, 2022).

[B9] Altmetric.com (2021b). Attention Sources Update Frequency and Collection Methods. Available online at: https://help.altmetric.com/support/solutions/articles/6000240275-attention-sources-update-frequency-and-collection-methods (accessed January 17, 2022).

[B10] Araújo R. F. (2020). Communities of attention networks: introducing qualitative and conversational perspectives for altmetrics. Scientometrics 124, 1793–1809. 10.1007/s11192-020-03566-7

[B11] Badenschier F. Wormer H. (2012). “Issue selection in science journalism: towards a special theory of news values for science news?,” in The Sciences' Media Connection—Public Communication and its Repercussions, eds S. Rödder, M. Franzen, and P. Weingart (New York, NY: Springer), 59–85. 10.1007/978-94-007-2085-5_4

[B12] Barata G. (2018). “The need to improve blogs qualitative data on altmetric,” in The 2018 Altmetrics Workshop: Science and The Public: Public Interactions with Science through the Lens of Social Media (London, UK). Available online at: http://altmetrics.org/wp-content/uploads/2018/04/altmetrics18_paper_10_Barata.pdf (accessed January 17, 2022).

[B13] Bar-Ilan J. Halevi G. Milojević S. (2019). Differences between altmetric data sources—a case study. J. Altmetric. 10.29024/joa.4

[B14] Belardinelli G. (2019). Gatekeepers in social networks: logics for communicative actions (Master's Thesis). University of Amsterdam. https://eprints.illc.uva.nl/id/eprint/1717/1/MoL-2019-20.text.pdf (accessed January 17, 2022).

[B15] Belli S. Gonzalo-Penela C. (2020). Science, research, and innovation infospheres in Google results of the Ibero-American countries. Scientometrics 123, 635–653. 10.1007/s11192-020-03399-4

[B16] Belli S. Mugnaini R. Baltà J. Abadal E. (2020). Coronavirus mapping in scientific publications: When science advances rapidly and collectively, is access to this knowledge open to society? Scientometrics 124, 2661–2685. 10.1007/s11192-020-03590-7 32836526 PMC7328881

[B17] BMBF (2018). FRONTAL—Research Rankings, Output Measurement, Recruitment of Young Researchers, Choice of Topics and Incentives for Care. Available online at: https://www.wihoforschung.de/en/frontal-2068.php (accessed January 17, 2022).

[B18] Boetto E. Fantini M. P. Gangemi A. Golinelli D. Greco M. Nuzzolese A. G.. (2021). Using altmetrics for detecting impactful research in quasi-zero-day time-windows: the case of COVID-19. Scientometrics 126, 1189–1215. 10.1007/s11192-020-03809-7 33424050 PMC7779112

[B19] Bornmann L. Haunschild R. (2018). Do altmetrics correlate with the quality of papers? A large-scale empirical study based on F1000Prime data. PLoS ONE 13, e0197133. 10.1371/journal.pone.0197133 29791468 PMC5965816

[B20] Bowman T. D. Niazi R. Ul Hassan S. (2019). “Science news and altmetrics: looking at EurekAlert!,” in The 2019 Altmetrics Workshop. BYOR: Bring Your Own Research (Stirling, UK). Available online at: http://altmetrics.org/wp-content/uploads/2019/10/Bowman_altmetrics19_paper_6.pdf (accessed January 17, 2022).

[B21] Bruns A. (2016). ““Random Acts of Journalism” Redux: news and social media,” in News Across Media: Production, Distribution and Consumption, eds J. Linaa, J. Jensen, M. Mortensen, and J. Ørmen (London: Routledge), 32–47. Available onlien at: https://www.taylorfrancis.com/chapters/edit/10.4324/9781315692456-8/random-acts-journalism-redux-news-social-media-axel-bruns (accessed January 17, 2022).

[B22] Cameron C. B. Nair V. Varma M. Adams M. Jhaveri K. D. Sparks M. A. (2016). Does academic blogging enhance promotion and tenure? A survey of US and Canadian medicine and pediatric department chairs. JMIR Med. Educ. 2, e10. 10.2196/mededu.4867 27731858 PMC5041355

[B23] Colavizza G. (2021). Meta-research on COVID-19: an overview of the early trends. *arXiv [preprint]*. https://arxiv.org/abs/2106.02961 (accessed January 17, 2022).

[B24] Colavizza G. Costas R. Traag V. A. van Eck N. J. van Leeuwen T. Waltman L. (2021). A scientometric overview of CORD-19. PLoS ONE 16, e0244839. 10.1371/journal.pone.0244839 33411846 PMC7790270

[B25] Colavizza G. Infelise M. Kaplan F. (2015). “Mapping the early modern news flow: an enquiry by robust text reuse detection,” in Social Informatics. SocInfo 2014, eds L. Aiello, and D. McFarland (Cham: Springer). 10.1007/978-3-319-15168-7_31

[B26] Dumas-Mallet E. Garenne A. Boraud T. Gonon F. (2020). Does newspapers coverage influence the citations count of scientific publications? An analysis of biomedical studies. Scientometrics 123, 413–427. 10.1007/s11192-020-03380-1

[B27] EC (2022). EMM—European Media Monitor. Available online at: https://ec.europa.eu/knowledge4policy/online-resource/emm-european-media-monitor_en (accessed January 17, 2022).

[B28] Fang Z. Costas R. (2020). Studying the accumulation velocity of altmetric data tracked by Altmetric.com. Scientometrics 123, 1077–1101. 10.1007/s11192-020-03405-9

[B29] Fassin Y. (2021). Research on Covid-19: a disruptive phenomenon for bibliometrics. Scientometrics 126, 5305–5319. 10.1007/s11192-021-03989-w 33994601 PMC8104038

[B30] Ferreira M. R. Ranjbar-Sahraei B. Costas R. (2020). “COVID-19 research in the news: Visualizing the sentiment and topics in science news about the pandemic,” in Leiden Madtrics. Available online at: https://leidenmadtrics.nl/articles/covid-19-research-in-the-news-visualizing-the-sentiment-and-topics-in-science-news-about-the-pandemic (accessed January 17, 2022).

[B31] Fleerackers A. Riedlinger M. Moorhead L. Ahmed R. Alperin J. P. (2021). Communicating scientific uncertainty in an age of COVID-19: an investigation into the use of preprints by digital media outlets. Health Commun. 10.1080/10410236.2020.1864892 33390033

[B32] Fraumann G. (2020). “What Lies behind Altmetrics Scores? Guidelines on How to Use Qualitative Approaches in Altmetrics,” in A Report from LIBER's Innovative Metrics Working Group. (LIBER)

[B33] Fraumann G. Zahedi Z. Costas R. (2015). “What do we know about altmetric.com sources? A study of the top 200 blogs and news sites mentioning scholarly output,” in The 2015 Altmetrics Workshop (Amsterdam, the Neherland).

[B34] Hauschke C. Cartellieri S. Heller L. (2018). Reference implementation for open scientometric indicators (ROSI). Res. Ideas Outcom. 4, e31656. 10.3897/rio.4.e31656

[B35] Haustein S. Bowman T. D. Costas R. (2015). “Communities of attention around scientific publications: Who is tweeting about scientific papers?,” in Social Media and Society 2015 International Conference (Toronto, Canada). Avialable online at: https://www.slideshare.net/StefanieHaustein/communities-of-attention-around-journal-papers-who-is-tweeting-about-scientific-publications (accessed January 17, 2022).

[B36] Homolak J. Kodvanj I. Virag D. (2020). Preliminary analysis of COVID-19 academic information patterns: a call for open science in the times of closed borders. Scientometrics 124, 2687–2701. 10.1007/s11192-020-03587-2 32836524 PMC7315688

[B37] Ioannidis J. P. A. Salholz-Hillel M. Boyack K. W. Baas J. (2021). The rapid, massive growth of COVID-19 authors in the scientific literature. Res. Soc. Open Sci. 8, 210389. 10.1098/rsos.210389 34527271 PMC8422596

[B38] Kwak H. Lee C. Park H. Moon S. (2010). “What is Twitter, a social network or a news media?,” in Proceedings of the 19th International Conference on World Wide Web (WWW '10). Available online at: http://www.ambuehler.ethz.ch/CDstore/www2010/www/p591.pdf (accessed January 17, 2022). 10.1145/1772690.1772751

[B39] Lomborg S. (2009). Navigating the blogosphere: towards a genre–based typology of weblogs. First Monday 14. 10.5210/fm.v14i5.2329

[B40] Lyu X. Costas R. (2021). How do academic topics shift across altmetric sources? A case study of the research area of big data. Scientometrics 123, 909–943. 10.1007/s11192-020-03415-7

[B41] Meschede C. Siebenlist T. (2018). Cross-metric compatability and inconsistencies of altmetrics. Scientometrics 115, 283–297. 10.1007/s11192-018-2674-1

[B42] NAS (2017). Communicating Science Effectively: A Research Agenda. Washington, DC: The National Academies Press. 28406600

[B43] Nuernbergk C. (2014). Follow-up communication in the blogosphere. Digit. Journal. 2, 434–445. 10.1080/21670811.2014.895520

[B44] Ørmen J. (2016). Googling the news. Digit. Journal. 4, 107–124. 10.1080/21670811.2015.1093272

[B45] Ortega J. L. (2018). Reliability and accuracy of altmetric providers: a comparison among Altmetric.com, PlumX and Crossref Event Data. Scientometrics 116, 2123–2138. 10.1007/s11192-018-2838-z

[B46] Ortega J. L. (2019). Availability and audit of links in altmetric data providers: link checking of blogs and news in Altmetric.com, Crossref Event data and PlumX. J. Altmetric. 2, 1–8. 10.29024/joa.14

[B47] Ortega J. L. (2020). Blogs and news sources coverage in altmetrics data providers: a comparative analysis by country, language, and subject. Scientometrics 122, 555–572. 10.1007/s11192-019-03299-2

[B48] Ortega J. L. Esquinas M. F. (2019). Evaluating Knowledge Transfer and Impact: Metrics, Procedures and Governance for Science and Innovation (Córdoba, Spain). Available online at: https://kti-conference2019.org/index_en.asp (accessed January 17, 2022).

[B49] Porter A. L. Zhang Y. Huang Y. Wu M. (2020). Tracking and mining the COVID-19 research literature. Front. Res. Metric. Anal. 5, 594060. 10.3389/frma.2020.594060 33870056 PMC8025982

[B50] Prados-Bo A. Casino G. (2021). Microbiome research in general and business newspapers: How many microbiome articles are published and which study designs make the news the most? PLoS ONE 16, e0249835. 10.1371/journal.pone.0249835 33836022 PMC8034714

[B51] Priem J. Taraborelli D. Groth P. Neylon C. (2010). Altmetrics: A Manifesto. Available online at: http://www.altmetrics.org/manifesto (accessed January 17, 2022).

[B52] Ross-Hellauer T. Tennant J. P. Banelyte V. Gorogh E. Luzi D. Kraker P.. (2020). Ten simple rules for innovative dissemination of research. PLoS Comput. Biol. 16, e1007704. 10.1371/journal.pcbi.1007704 32298255 PMC7161944

[B53] Scott D. T. (2008). “Tempests of the blogosphere: Presidential campaign stories that failed to ignite mainstream media,” in Digital Media and Democracy: Tactics in Hard Times. eds M. Boler, 271–300. Available online at: http://web.mit.edu/comm-forum/legacy/mit4/papers/scott.pdf (accessed January 17, 2022).

[B54] Shema H. Bar-Ilan J. Thelwall M. (2014). Do blog citations correlate with a higher number of future citations? Research blogs as a potential source for alternative metrics. J. Assoc. Inf. Sci. Technol. 65, 1018–1027. 10.1002/asi.23037

[B55] Shema H. Bar-Ilan J. Thelwall M. (2015). How is research blogged? A content analysis approach. J. Assoc. Inf. Sci. Technol. 66, 1136–1149. 10.1002/asi.23239

[B56] Tunger D. Clermont M. Meier A. (2018). “Altmetrics: state of the art and a look into the future,” in Scientometrics. eds M. Jibu, and Y. Osabe. 10.5772/intechopen.76874

[B57] Turki H. Hadj Taieb M. A. Shafee T. Lubiana T. Jemielniak D. Aouicha M. B.. (2021). Representing COVID-19 information in collaborative knowledge graphs: the case of Wikidata. Semant. Web. 13, 233–264. 10.3233/SW-210444

[B58] van der Sluis F. Glassey R. J. van den Broek E. L. (2012). “Making the news interesting: understanding the relationship between familiarity and interest,” in Proceedings of the 4th Information Interaction in Context Symposium, 314–317 (Nijmegen, The Netherlands). 10.1145/2362724.2362783

[B59] WHO (2020). WHODirector-General's Opening Remarks at the Media Briefing on COVID-19-−*11 March 2020*. Available online at: https://www.who.int/director-general/speeches/detail/who-director-general-s-opening-remarks-at-the-media-briefing-on-covid-19-−11-march-2020 (accessed January 17, 2022).

[B60] WHO (2022). Coronavirus Disease (COVID-19) Pandemic. Available online at: https://www.who.int/emergencies/diseases/novel-coronavirus-2019 (accessed January 17, 2022).

[B61] Woolston E. Brown C. (2018). Why science blogging still matters. Nature 554, 135–137. 10.1038/d41586-018-01414-6 32094863

[B62] WordPress (2022). A live look at Activity Across WordPress.com. Available online at: https://wordpress.com/activity/ (accessed January 17, 2022).

[B63] Zahedi Z. Costas R. (2018). General discussion of data quality challenges in social media metrics: extensive comparison of four major altmetric data aggregators. PLOS ONE 13, e0197326. 10.1371/journal.pone.0197326 29772003 PMC5957428

[B64] Zhang L. Zhao W. Sun B. Huang Y. Glänzel W. (2020). How scientific research reacts to international public health emergencies: a global analysis of response patterns. Scientometrics 124, 747–773. 10.1007/s11192-020-03531-4 32836522 PMC7282204

